# Dataset of the relationship between authentic virtual reality experiences and tourists’ visiting intentions

**DOI:** 10.1016/j.dib.2024.111129

**Published:** 2024-11-13

**Authors:** Thi Bich Thuy Nguyen, Quoc Vinh Pham-Le, Ngoc Tuan Chau

**Affiliations:** a Faculty of Business Administration, University of Economics, The University of Danang, Vietnam; b Faculty of Statistics and Informatics, University of Economics, The University of Danang, Vietnam

**Keywords:** Tourism destination, Virtual reality, Destination marketing, Authentic experience, Visiting intention, Danang, Vietnam

## Abstract

After the pandemic, the demand for a rapid recovery of tourism has led to increased intense competition among destinations, posing a considerable challenge for tourism managers and destination marketers worldwide. This situation necessitates a constant seek for new, unique, and more attractive methods to promote tourism destinations. Virtual Reality (VR) emerges as a promising solution with the potential to significantly transform destination marketing activities. This article presents a dataset exploring the relationship between VR authentic experience, cognitive state, affirmative state, and behavioral visiting intentions of potential tourists experiencing VR at the destination of Danang, Vietnam. The dataset includes 359 survey samples collected from potential tourists in four major cities in Vietnam including Thanh Hoa, Vinh, Hanoi, and Phu Quoc. This dataset is crucial for providing insights into how VR experiences impact the visiting intentions of potential tourists towards a specific destination. The shared dataset aims to lay the foundation for future comparative research, expanding knowledge about the influence of VR experiences specifically and smart tourism technologies in general on tourists’ visiting intentions.

Specifications Table

**Table:** 

Subject	Management of Technology and Innovation
Specific subject area	Virtual Reality experience and visiting intention
Data format	Raw, Analyzed
Type of data	Table, Figure, MS Excel file
Data collection	Data was collected during the period from April 2023 to July 2023. The participants were selected using a convenient sampling method. A total of 405 responses were received. Data cleaning and examination processes were conducted to address the missing values, outliers, and normality, leading to the deletion of 46 cases with unanswered questions, missing data, and outliers. The final 359 usable responses were retained for statistical analysis.
Data source location	City/Town/Region: Danang Country: Vietnam Coordinates: 16.0544° N, 108.2022° E
Data accessibility	Repository name: Mendeley Data Data identification number: 10.17632/2tg7ffdr56.1 Direct URL to data: 10.17632/2tg7ffdr56.1

## 1 Value of the Data

• Accessing the raw data is readily available to researchers, as it is conveniently prepared for their utilization.
• The data holds the promise of providing valuable insights into tourists’ behavioral intentions and the use of VR technology in destination marketing, given the potential development of the tourism industry after the COVID-19 outbreak.
• The dataset presents an opportunity for conducting comparative studies across different tourism destinations [1]. This invaluable resource can serve as a launching pad for investigating the influence of VR experience on tourists’ visiting intention within the Stimulus-Organism-Response (SOR) model across various tourism destinations [2].
• The dataset stands as a crucial asset for researchers as well as destination management organizations and managers who are interested in the areas of the use of technology in destination management and marketing and the effect of technology experience on the tourist’ visiting intention.
• The use of structural model analysis for the dataset provides various methodological and theoretical contributions. This enhances the accuracy and comprehensiveness of the analysis, leading to valuable insights into the relationship between VR experience and tourists’ visiting intention.

## 2 Data Description

This data article aims to provide a detailed examination of quantitative data regarding the influence of authentic VR experience on the cognitive state and affirmative state of potential tourists, and subsequently, on their visiting intentions. The data was collected through a survey, featuring a questionnaire with 30 items utilizing a five-point Likert scale to evaluate participants’ responses. This survey instrument comprises three first-order and eight second-order research constructs, along with 30 measurement itemsinspired by previous research in similar areas. Table 1 presents these eight second-order constructs along with their associated measurement items and reference sources. Responses were numerically coded as follows: 5 for “strongly agree,” 4 for “agree,” 3 for “neutral,” 2 for “disagree,” and 1 for “strongly disagree.”

**Table 1:** Constructs and measurement items.

Constructs	Measurement items		References
Presence (PRE)	PRE1	I feel like I'm in the middle of the city when using VR on the Danang tourism destination website.	[3]
	PRE2	I feel like my actual location is in Danang when experiencing VR on the Danang tourism destination website.	
	PRE3	It seems like I am truly present in the virtual environment rendered by VR.	
	PRE4	I feel that I can move around to explore the scenery and participate in tourist activities at the destination when using VR on the Danang tourism destination website.	
Aesthetics (AES)	AES1	The website platform interface is designed sharply.	[4]
	ASE2	The website platform interface design is beautiful.	
	AES3	The layout of the website platform interface is attractive.	
	AES4	The design of the website platform interface is artistic.	
Usefulness (USE)	USE1	Using VR on the Danang tourism destination website brings benefits to me.	[5]
	USE2	Using VR on the Danang tourism destination website helps me gain more knowledge about this tourism destination.	
	USE3	Using VR on the Danang tourism destination website helps me make friends/connect with others who have watched videos.	
	USE4	VR on the Danang tourism destination website is very useful in improving the efficiency of receiving destination information.	
Ease of Use (EAS)	EAS1	It is very easy to use VR on the Danang tourism destination website.	[5]
	EAS2	I can easily use VR to explore what I want to know about the Danang tourism destination.	
	EAS3	I can use VR on the Danang tourism destination website easily from anywhere.	
Enjoyment (ENJ)	ENJ1	Using VR on the Danang tourism destination website makes me feel interested.	[6]
	ENJ2	Using VR on the Danang tourism destination website makes me feel excited.	
	ENJ3	Using VR on the Danang tourism destination website makes me feel fun.	
	ENJ4	Using VR on the Danang tourism destination website makes me feel happy.	
Emotional Involvement (EMO)	EMO1	I am completely drawn to engage in using VR on the Danang tourism destination website.	[7]
	EMO2	I am very impressed with the activities that can be performed when using VR on the Danang tourism destination website.	
	EMO3	I feel immersed in the virtual environment from the VR experience on the Danang tourism destination website.	
Flow State (FLOW)	FLOW1	During the use of VR on the Danang tourism destination website, I feel completely captivated.	[8]
	FLOW2	During the use of VR on the Danang tourism destination website, I feel that time passes by very quickly.	
	FLOW3	During the use of VR on the Danang tourism destination website, I temporarily forget all external concerns.	
	FLOW4	During the use of VR on the Danang tourism destination website, I forget where I am.	
Visit Intention (INT)	INT1	I intend to visit Danang after experiencing this destination through VR on the Danang tourism destination website.	[9]
	INT2	I plan to visit Danang after experiencing this destination through VR on the Danang tourism destination website.	
	INT3	I feel ready to visit Danang after experiencing this destination through VR on the Danang tourism destination website.	
	INT4	I am willing to spend money and time to visit Danang after experiencing this destination through VR on the Danang tourism destination website.	

The survey was conducted using face-to-face interviews with tourists in four selected major cities in Vietnam: Hanoi, Thanh Hoa, Vinh, and Phu Quoc. This selection was based on rigorous considerations of the sample's representativeness and generalizability. These four locations are situated in different regions of Vietnam: the north, the central, and the south. Additionally, the selection took into account the unique tourism products of each city. Specifically, Hanoi, the capital, is renowned for its historical and cultural heritage, while Phu Quoc is an island city known for sea travel. Thanh Hoa and Vinh are famous for their local foods and beautiful inland landscapes. The diverse sample of tourists, with their varying tourism preferences, provides valuable perspectives on VR experience and visiting intentions. To have the most representative sample, the study secondly employs a conditional random sampling method, with the key condition being that potential tourists have never visited Danang city and are capable of using VR technology, meaning they own VR-enabled devices such as headsets or smartphones. From an initial random sample of 680 individuals, 505 participants met these criteria. These 505 individuals were subsequently approached for participation in the survey.

The accumulated raw data was stored in an Excel file comprising 360 rows and 36 columns. The initial row delineates the sample characteristics and the measurement items.Rows 2 to 360 encapsulate the data pertraining to each of the 359 participants. Columns 1 to 6 capture data related to the demographic characteristics of the survey participants, while columns 7 to 36 encompass information about the research variables.

The quantitative data analysis follows a comprehensive process, including data preparation, descriptive statistics, measurement instrument assessment and validation, and hypothesis testing. This article provides summarizes the results of this process through seven tables and one figure.

Table 2 provides details about the demographic characteristics of the survey respondents who have experienced VR on the Danang tourism destination website. This information includes gender distribution, marital status, income levels, age group, and the number of times respondents visited domestic and international tourism destinations in 2023. The data indicate that the majority of respondents are female. More than half of being married. A significant portion of respondents have a monthly income of 5 to 10 million VND. Nearly half of the respondents belong to the age group from 31 to 55, and the majority of them have visited 1 to 3 tourism destinations at the time of this survey.

**Table 2:** Demographic characteristics of the sample.

Category	Description	Frequency	Percentage
Gender	Male	84	23.4
	Female	275	76.6
Marital status	Single	154	42.9
	Married	199	55.4
	Other	6	1.7
Income (VND)	<5 million	106	29.5
	5 – <10 million	173	48.2
	10 - <20 million	67	18.7
	≥20 million	13	3.6
Age group	18–30	172	47.9
	31–55	180	50.1
	>55	7	1.9
Number of times traveling (domestic and international) this year	0	127	35.4
	1–3	192	53.5
	4–6	20	5.6
	>6	20	5.6

Table 3 presents the mean, standard deviation, and normality assessment results for all measurement items through the analysis of Skewness and Kurtosis values. The evaluation used critical values of ±2.58 at a 1 % significance level [10]. The data show that all measurement items have Skewness values ranging from −0.488 to 0.265 and Kurtosis values ranging from −1.339 to −0.248. These values are within the acceptable range for normality. As a result, the data of all measurement items are normally distributed.

**Table 3:** Descriptive statistics and normality assessment for measurement items.

Construct	Item	N	Mean	Std. Dev	Skewness	Kurtosis
Presence (PRE)	PRE1	359	4.25	0.560	0.001	−0.372
	PRE2	359	4.20	0.568	−0.007	−0.248
	PRE3	359	4.28	0.588	−0.150	−0.540
	PRE4	359	4.28	0.617	−0.258	−0.620
Aesthetics (AES)	AES1	359	4.32	0.513	0.265	−0.851
	ASE2	359	4.32	0.561	−0.083	−0.659
	AES3	359	4.31	0.522	0.194	−0.754
	AES4	359	4.25	0.597	−0.152	−0.513
Usefulness (USE)	USE1	359	3.96	0.635	0.030	−0.511
	USE2	359	4.01	0.675	−0.017	−0.791
	USE3	359	4.05	0.623	−0.034	−0.419
	USE4	359	4.01	0.707	−0.059	−0.853
Ease of Use (EAS)	EAS1	359	4.13	0.629	−0.109	−0.522
	EAS2	359	4.23	0.664	−0.296	−0.775
	EAS3	359	4.15	0.643	−0.148	−0.626
Enjoyment (ENJ)	ENJ1	359	4.06	0.629	−0.045	−0.471
	ENJ2	359	3.98	0.670	0.022	−0.763
	ENJ3	359	4.04	0.669	−0.048	−0.758
	ENJ4	359	4.05	0.632	−0.040	−0.491
Emotional Involvement (EMO)	EMO1	359	4.17	0.599	−0.083	−0.369
	EMO2	359	4.05	0.650	−0.049	−0.623
	EMO3	359	4.06	0.688	−0.083	−0.881
Flow State (FLOW)	FLOW1	359	4.05	0.669	−0.117	−0.566
	FLOW2	359	4.05	0.689	−0.062	−0.888
	FLOW3	359	4.02	0.717	−0.079	−0.910
	FLOW4	359	4.02	0.724	−0.078	−0.957
Visit Intention (INT)	INT1	359	4.41	0.604	−0.488	−0.640
	INT2	359	4.32	0.630	−0.375	−0.674
	INT3	359	4.25	0.710	−0.392	−0.960
	INT4	359	4.13	0.784	−0.229	−1.339

Tables 4 and 5 summarize the results of non-response bias and common method bias tests respectively. Non-response bias was assessed by comparing the patterns of “early” and “late” respondents on the study variables. The first 30 responses and the last 30 responses were selected for a two-sample *t*-test to compare the mean differences. The results indicate no significant differences between earlier and later responses at a 95 % confidence interval for the chosen variables. Common method bias was tested using Harman's single-factor method. The results reveal the presence of eight factors with eigenvalues greater than 1, accounting for 80.105 % of the variances in the measures. The greatest factor explains only 38.828 % of the variance, which is less than 50 % threshold, indicating no significant common method bias. Additionally, no single factor emerged to represent the variance among all the measurement items, further suggesting the absence of significant bias due to the research method used [11].

**Table 4:** Independent sample *t*-test for non-response bias.

Dimension	t	df	p	Mean			Std. Error Difference
				Earlier	Later	Difference	
Mean of True Experience	1.725	58	0.104	4.421	4.225	0.196	0.114
Mean of Cognitive	0.000	58	0.125	4.233	4.233	0.000	0.124
Mean of Affirmative	0.077	58	0.744	4.142	4.133	0.009	0.118
Mean of Intention	0.311	58	0.965	4.542	4.500	0.042	0.134

**Table 5:** Common method bias test – Total variance explained.

Component	Initial Eigenvalues			Extraction Sums of Squared Loadings		
	Total	% of Variance	Cumulative %	Total	% of Variance	Cumulative %
1	11.648	38.828	38.828	11.648	38.828	38.828
2	3.580	11.935	50.763	3.580	11.935	50.763
3	2.002	6.675	57.437	2.002	6.675	57.437
4	1.700	5.667	63.104	1.700	5.667	63.104
5	1.565	5.216	68.320	1.565	5.216	68.320
6	1.378	4.594	72.914	1.378	4.594	72.914
7	1.155	3.851	76.765	1.155	3.851	76.765
8	1.002	3.340	80.105	1.002	3.340	80.105

Table 6 presents the results of internal consistency reliability and convergent validity. The collected data satisfies the criteria for internal consistency reliability, with all constructs exhibiting Composite Reliability (CR) values surpassing 0.7. Additionally, Cronbach's Alpha values also surpass the 0.7 threshold [12]. Convergent validity was assessed through examination of outer loadings and the average variance extracted (AVE). The results illustrate that all measurement items exhibit outer loadings surpassing the 0.7 threshold. The AVE, representing common variance within a construct, was determined to be no less than 0.5 for each of the eight constructs. These findings indicate satisfactory convergent validity across all constructs [12].

**Table 6:** A summary of reliability assessment and convergent validity.

Construct	Item	Outer loandings	Cronbach's Alpha	Composite Reliability (CR)	Average Variance Extracted (AVE)
Presence	PRE1	0.880	0.873	0.913	0.724
	PRE2	0.841			
	PRE3	0.850			
	PRE4	0.832			
Aesthetics	AES1	0.871	0.900	0.930	0.769
	ASE2	0.879			
	AES3	0.862			
	AES4	0.897			
Usefulness	USE1	0.905	0.899	0.930	0.769
	USE2	0.895			
	USE3	0.830			
	USE4	0.875			
Ease of Use	EAS1	0.896	0.873	0.922	0.797
	EAS2	0.889			
	EAS3	0.894			
Enjoyment	ENJ1	0.907	0.911	0.937	0.789
	ENJ2	0.866			
	ENJ3	0.904			
	ENJ4	0.876			
Emotional Involvement	EMO1	0.920	0.911	0.944	0.849
	EMO2	0.909			
	EMO3	0.935			
Flow State	FLOW1	0.910	0.934	0.953	0.834
	FLOW2	0.911			
	FLOW3	0.926			
	FLOW4	0.907			
Visit Intention	INT1	0.893	0.931	0.951	0.829
	INT2	0.916			
	INT3	0.919			
	INT4	0.913			

Table 7 presents results of discriminant validity using Heterotrait-Monotrait (HTMT) ratio criteria. From the perspective of HTMT ratio, the results of the valid values for each pair of variables indicate that such values for each first-order variable are all below 0.9, even lower than the threshold of 0.85 [12,13]. Therefore, the criterion for the discriminant value between variables is established.

**Table 7:** Discriminant validity using Heterotrait-Monotrait Ratio (HTMT) of correlation criterion.

	(1)	(2)	(3)	(4)	(5)	(6)	(7)	(8)
AESTHETICS (1)								
EASE OF USE (2)	0.202							
EMOTIONAL (3)	0.450	0.404						
ENJOYMENT (4)	0.348	0.565	0.376					
FLOW STATE (5)	0.549	0.327	0.555	0.437				
PRESENCE (6)	0.633	0.298	0.409	0.320	0.526			
USEFULNESS (7)	0.222	0.557	0.352	0.528	0.370	0.273		
VISIT INTENTION (8)	0.501	0.495	0.562	0.588	0.668	0.394	0.561	

**Fig. 1:**
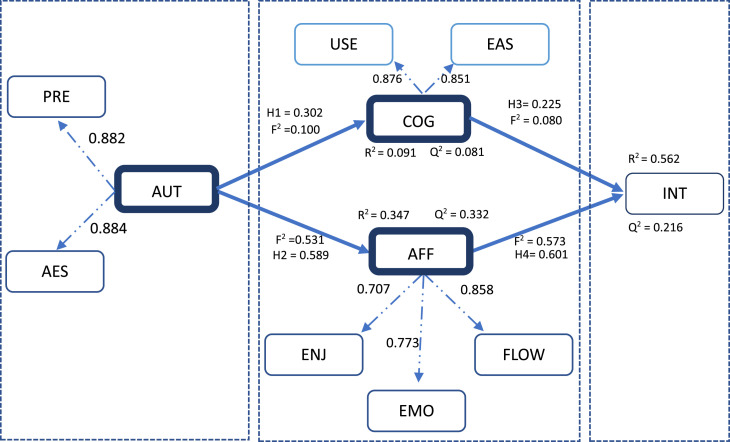
Statistical relationship between constructs.

Fig. 1 presents the results of hypothesis testing and structural model. Determining an appropriate sample size for structural model analysis lacks a universally accepted method, so researchers often rely on practical rules of thumb. For instance, [14] recommend a minimum ratio of 10 observations per estimated parameter, while [15] propose a ratio of 5 to 1. This study employs the “Invert square root” approach [16] for the technical minimum sample size. Based on this approach, the minimum sample size for this study was calculated at 316. As a result, the real sample size of 359 participants with 30 observed variables in this study is deemed appropriate for structural model analysis.

The initial hypotheses, tested in the positive direction, are accepted with a significance level of 0.05 [14]. Running bootstrapping with 10,000 subsamples showed that *p*-values are significant and less than the 0.05 threshold. Multicollinearity testing revealed VIF values all below 3, indicating no multicollinearity issues in the model [12,17].

The results also indicate that R^2^ values for affirmative state and visiting intention at 0.347 and 0.562, respectively. The R^2^ value of cognitive state is 0.091, which is greater than 0.02, ensuring the explanatory power of variables in the model [18]. Additionally, the Q^2^ coefficients for the cognitive state, affirmative state, and visiting intention achieve stable values (greater than 0.2), indicating good predictive capability of the model [17].

The cross-validated predictive ability test (CVPAT) was conducted to evaluate the robustness or predictive power of the PLS-SEM model [19]. Table 8 presents the comparative results of the loss values between the PLS-SEM model and the Indicator Average (IA) method. The comparison is conducted across three latent variables (LVs): Affirmative, Cognitive, and Visit Intention. The findings reveal that the PLS-SEM model exhibits significantly lower loss levels compared to the IA method for all LVs. This is evidenced by the substantial difference in loss values between PLS-SEM and IA, along with a p-value less than 0.05 for all LVs. Therefore, the PLS-SEM model demonstrates superior robustness over the IA method in this study. These results confirm that PLS-SEM provides greater accuracy in explaining complex or nonlinear relationships among LVs.

**Table 8:** CVPAT LVs summary: PLS-SEM vs. indicator average.

Latent variable	PLS Loss	IA Loss	Average Loss Difference	*t-*value	*p-*value
Affirmative	0.808	1.006	−0.198	5.576	0.000
Cognitive	0.947	1.005	−0.058	2.355	0.019
Visit Intention	0.390	0.472	−0.082	5.185	0.000
Overall	0.653	0.768	−0.115	5.471	0.000

Table 9 presents the comparative loss values between the PLS-SEM model and the linear model (LM) across three LVs: Affirmative, Cognitive, and Visit Intention. These values are compared both individually and in aggregate. The results indicate that there is no statistically significant difference between the LM and the PLS-SEM models for any of the LVs. This is evidenced by the minimal difference in loss values and a *p*-value greater than 0.05 across all LVs. Therefore, the data explanatory accuracy of both models is comparable. Given the absence of a significant difference in accuracy between the two models, the PLS-SEM model is deemed sufficiently accurate for explaining the relationships among the variables in this study.

**Table 9:** CVPAT LVs summary: PLS-SEM vs. Linear model.

Latent variable	PLS Loss	LM Loss	Average Loss Difference	*t-*value	*p-*value
Affirmative	0.808	0.809	−0.001	0.281	0.779
Cognitive	0.947	0.949	−0.002	0.331	0.741
Visit Intention	0.390	0.387	0.003	0.847	0.397
Overall	0.653	0.652	0.001	0.361	0.719

## 3 Experimental Design, Materials and Methods

The study primarily utilized quantitative research methods, collecting data through a structured questionnaire. All measurement items were derived from prior English-language studies to ensure the questionnaire's logical structure, comprehensibility, and suitability for the Vietnamese research context while maintaining meaningful equivalence. The scales were refined based on discussions with five experts in the field of tourism and feedback from five potential tourists who experienced VR on the Danang tourism destination website. The refined instrument was pilot tested with forty tourists in the four selected cities (ten for each) to assess the reliability of the measurement scale. These tourists were asked to complete the questionnaire, provide feedback on any unclear questions and offer suggestions for improvement. Based on their feedback, minor revision was made, including rewording some statements to enhance clarity and comprehensibility.

The sample was selected using a convenient sampling method. The minimum sample size was determined to be 150, based on an observation-to-variable ratio of 5:1 [11]. Respondents were potential tourists who had never visited Danang. Participants were aged 18 and above and were invited to experience a 3 to 5-minute virtual tour of attractions in Danang using the VR headset Gear Shinecon G10 and various Android and iOS smartphones. This experience was facilitated through the platform “vr360.danangfantasticity.com.”

The measurement model in the study followed a result-cause structure based on the SOR model [20]. The authentic experience, cognitive state, and affirmative state are three higher-order latent variables that act as causes for first-order variables: aesthetics, presence, usefulness, ease of use, enjoyment, emotional involvement, and flow state. These first-order variables were measured through 30 observed measurement items.

A two-stage approach was employed to validate the research model [21]. In the first stage, first-order variables and measurement items were assessed for reliability, convergence validity, and discriminant validity using Statistical Package for the Social Sciences (SPSS) 28 software. In the second stage, structural model testing was conducted through bootstrapping analysis, hypothesis testing, multicollinearity examination, effect size (f^2^), explanatory power (R^2^), and model prediction (Q^2^). Partial Least Squares Structural Equation Modeling (PLS-SEM) was deemed suitable for examining complex models using higher-order latent variables [17]. SmartPLS 4.0 software was utilized for a structured analysis.

## Limitations

The most critical limitation of the dataset and the research is the restricted research population. While the insights gained from the sample of 359 potential domestic tourists in four major cities in Vietnam provide valuable information about the relationships between authentic VR experience, cognitive state, affirmative state, and visiting intention, there are potential limitations in generalizing these research findings to the broader population of Vietnam.

Furthermore, this study exclusively utilizes the online VR tourism platform provided by the city of Danang to evaluate the proposed research model. This implies that the findings may not be representative of other destinations in Vietnam that also implement VR tourism. The sample's representativeness is further constrained by its uneven distribution of the sample characteristics with the majority of the respondents are female, younger, and middle-class income earners.

To address these limitations, researchers interested in utilizing this dataset for future investigations should consider enhancing the sample size by including a more diverse range of participants. Specifically, incorporating more males and individuals from different age groups and income levels, and recruiting participants from other cities in Vietnam or even from other countries, would provide a more holistic picture of the influence of authentic VR experience on cognitive and affirmative states, and subsequently on the visiting intentions of potential tourists. Additionally, conducting comparative studies using datasets from other contexts could offer further insights into the subject matter. Such comparative studies contribute to a more nuanced and robust interpretation of the findings, thereby advancing the literature in this research area.

## Ethics Statement

We sought the consent of all survey participants, which was obtained through a statement detailing the survey's purpose and objectives. Additionally, we took measures to guarantee the anonymity of respondents, ensuring that no personal information could be linked back to them.

The study was conducted in accordance with the Declaration of Helsinki and approved by the Ethics Committee of University of Economics - The University of Danang, Vietnam (Approval code 93/QD-DHKT, Approval date: January 14th, 2022).

## CRediT Author Statement

**Thi Bich Thuy Nguyen:** Conceptualization, Investigation, Original draft preparation; **Quoc Vinh Pham-Le:** Methodology, Data collection, Validation, Original draft preparation; **Ngoc Tuan Chau:** Data analysis, Writing – review and editting, final paper.

## Data Availability

Mendeley DataDataset of the relationship between authentic virtual reality experiences and tourists’ visiting intentions of a tourism destination (Original data).

## References

[1] Ouerghemmi C., Ertz M., Bouslama N., Tandon U. (2023). The impact of Virtual Reality (VR) tour experience on tourists’ Intention to visit. Information.

[2] Calisto M.L., Sarkar S. (2024). A systematic review of virtual reality in tourism and hospitality: the known and the paths to follow. Int. J. Hosp. Manag..

[3] Wirth W., Hartmann T., Bocking S., Vorderer P., Klimmt C., Schramm H., Saari T., Laarni J., Ravaja N., Gouveia F.R. (2007). A process model of the formation of spatial presence experiences. Media Psychol..

[4] Pallud J., Straub D.W. (2014). Effective website design for experience-influenced environments: the case of high culture museums. Inf. Manag..

[5] Huang Y.C., Backman S.J., Backman K.F., Moore D. (2013). Exploring user acceptance of 3D virtual worlds in travel and tourism marketing. Tour. Manag..

[6] Guo Y., Barnes S. (2011). Purchase behavior in virtual worlds: an empirical investigation in Second Life. Inf. Manag..

[7] Holsapple C.W., Wu J. (2007). User acceptance of virtual worlds: the hedonic framework. ACM SIGMIS Database: Database Adv. Inf. Syst..

[8] Siau K., Nah F.F.H., Mennecke B.E., Schiller S.Z. (2010). Co-creation and collaboration in a virtual world: a 3D visualization design project in Second Life. J. Database Manag..

[9] Tussyadiah I.P., Wang D., Jung T.H., Tom D.M.C. (2018). Virtual reality, presence, and attitude change: empirical evidence from tourism. Tour. Manag..

[10] Hair J., Hair J.F., Hult G.T.M., Ringle C.M., Sarstedt M. (2021).

[11] J.F. Hair, W.C. Black, B.J. Babin, R.E. Anderson, R.L. Tatham, Multivariate Data Analysis, 2010: Upper Saddle River, New Jersey, United States.

[12] Ringle C.M., Sarstedt M., Sinkovics N., Sinkovics R.R. (2023). A perspective on using partial least squares structural equation modelling in data articles. Data Br..

[13] Henseler J., Ringle C.M., Sarstedt M. (2015). A new criterion for assessing discriminant validity in variance-based structural equation modeling. J. Acad. Mark. Sci..

[14] Schreiber J.B., Nora A., Stage F.K., Barlow E.A., King J. (2006). Reporting structural equation modeling and confirmatory factor analysis results: a review. J. Educ. Res..

[15] Bentler P.M., Chou C.P. (1987). Practical issues in structural modeling. Sociol. Methods Res..

[16] Kock N., Hadaya P. (2018). Minimum sample size estimation in PLS-SEM: the inverse square root and gamma-exponential methods. Inf. Syst. J..

[17] Hair J.F., Risher J.J., Sarstedt M., Ringle C.M. (2019). When to use and how to report the results of PLS-SEM. Eur. Bus. Rev..

[18] Cohen J. (2013).

[19] Liengaard B.D., Sharma P.N., Hult G.T.M., Jensen M.B., Sarstedt M., Hair J.F., Ringle C.M. (2021). Prediction: coveted, yet forsaken? Introducing a cross-validated predictive ability test in partial least squares path modeling. Decis. Sci..

[20] Mehrabian A., Russell J.A. (1974).

[21] Sarstedt M., Hair J.F., Cheah J.H., Becker J.M., Ringle C.M. (2019). How to specify, estimate, and validate higher-order constructs in PLS-SEM. Austral. Mark. J..

